# The opioid crisis fuelled by health systems: how will future physicians fare?

**DOI:** 10.1192/bji.2023.12

**Published:** 2023-08

**Authors:** Mark Mohan Kaggwa, Jeremy Devine, Sheila Harms

**Affiliations:** 1 Department of Psychiatry and Behavioural Neurosciences, McMaster University, Hamilton, Ontario, Canada. Email: kmarkmohan@gmail.com; 2 Department of Psychiatry, Faculty of Medicine, Mbarara University of Science and Technology, Mbarara, Uganda

**Keywords:** Opioid crisis, addiction, opioid use disorder, tramadol, pharmakon

## Abstract

The opioid crisis continues to affect many areas worldwide, raising questions regarding prescribing indications. There is no consensus on negotiating the need for pain relief and the potential for medically prescribed opioid-related harm/addiction. These issues present an enormous educational challenge to physicians in training, particularly those whose mandate is to understand and respond to varying forms of pain. This article examines the perspectives and educational challenges faced by two psychiatry residents from different parts of the globe during the crisis. Is the educational experience of future psychiatrists sufficient to prepare them for the responsibilities that lie ahead?

In reading about the opioid crisis and its global impact on healthcare, conflicting themes are often portrayed. Frequently we encounter descriptions of a failed healthcare system leaving affected victims struggling with profound substance misuse left abandoned, stigmatised and systemically forced into an increasingly marginalised existence. Placed in contrast to this perspective is the pharmaceutical industry, represented by a handful of physician prescribers, vilified for unethical practice and benefitting in the wake of others’ misfortune. In this article, we propose to look at the opioid crisis differently, particularly as an educational problem for two psychiatry trainees (M.M.K. and J.D.) who represent learning viewpoints from different parts of the globe. As an educational case, this reflection brings together two diverse trainee perspectives – one from Uganda and one from Canada – where we are attempting to understand the opioid crisis precisely as an educational problem that crosses borders. We explore the opioid crisis as a focused foray into the various determinants of health that affect it and how medical education shapes an understanding of the opioid problem.

## The cases that brought us together

The discussion between us started with two cases. The first had to do with Mr A, a 35-year-old Ugandan man with a successful business and no previous history of substance use, who presented to a Ugandan psychiatric out-patient setting with several complaints about sexual dysfunction in the context of tramadol use for the past 2 years. Two years earlier, Mr A had experienced abdominal pain unrelieved by common analgesics. Despite a negative investigation for the cause of Mr A's pain, a clinician prescribed a short course of intravenous (IV) tramadol for temporary relief. In addition to pain control, the patient reported a feeling of euphoria as well as withdrawal symptoms without consistent access to tramadol. The patient's tramadol use increased to the point where he would present to clinics fabricating symptoms of severe abdominal pain in the hopes of being given IV tramadol. He later learned how to self-administer injections and bought tramadol in bulk, as it was easily available in Ugandan pharmacies. Mr A's sexual dysfunction worsened, leading to intense psychological distress, depressive symptoms and suicidal thoughts. Neither traditional healing practices nor Western medicine provided successful solutions for his sexual dysfunction, which increased his reliance on tramadol.

In the Canadian training context for learning psychiatry, addressing psychic pain and distress is a common problem identified by patients and physicians. The resident (J.D.) had numerous clinical encounters where opioids were appropriately prescribed to those receiving end-of-life care but also to those with vague complaints of pain, often subjectively linked to difficult life circumstances. Some patients appeared to be receiving opioids to relieve emotional and psychological pain associated with difficult life circumstances. In these situations, he was worried that underlying issues leading to reports of pain would be left unrecognised, uninvestigated or, worse, iatrogenically subdued by potent medications, leaving patients less able to engage on the road to recovery. He wondered whether the pain of coping with life's struggles could take on something other than a demand for palliation, particularly in psychiatry.

## The Ugandan learner perspective (M.M.K.)

Throughout my work with Mr A, his suffering was understood as secondary to an opioid use disorder with sexual dysfunction as a side-effect. The available African medical literature provided little information about the incidence and prevalence rates of opioid misuse or secondary sexual dysfunction associated with opioid use in Uganda. A local survey reported that many opioids, such as morphine, tramadol and pethidine, are easily available to the Ugandan public.^1^ Further research in the grey literature revealed that in 2004, Ugandan laws on opioid prescriptions were adjusted, allowing most health workers (i.e. nurses, doctors and clinical officers) to prescribe opioids because of human resource shortages.^2^ The World Health Organization (WHO) also removed most opioids from its Essential Medicines List. Specifically, it declined to add tramadol to the scheduled list of medications when it was under review.^3^ However, opioids, including tramadol, are incorporated into Uganda's essential medicines list and are widely available despite the WHO changes.^4^ Despite the problems reported by patients, tramadol was not included as a drug of misuse in the WHO list even though it is a centrally acting synthetic opioid analgesic. This information created a tautological problem in education.

## The North American learner perspective (J.D.)

Looking to understand the historical context of the opioid crisis, using tramadol misuse as a clinical example in North America, I located information suggesting that it was initially introduced in Europe and found to be significantly less potent when injected than morphine. However, widespread reports of tramadol misuse allowed for discovery of a potent metabolite with similar misuse potential compared with other high-potency opioids.^5^ Despite this phenomenon, tramadol was introduced into the North American marketplace and resulted in a 250% increase in tramadol-related emergency department visits from 2005 to 2011.^5^ These statistics show that the demands for pain relief are partly driving the consumption patterns. But the thorny question about which type of pain warrants the use of narcotics is front and centre of the educational conundrum. Rights-based discussions reflected in high-profile medical journals have reported that patients struggling with diverse clinical pain syndromes have the right to pain elimination. For example, an article in *Anesthesia & Analgesia* stated ‘the unreasonable failure to treat pain is poor medicine, unethical practice, and is an abrogation of a fundamental human right’, thereby advocating for a conceptualisation of complex chronic pain syndromes as a type of unique disease entity.^6^ The main educational problem suggested in this statement would have physicians treat pain at all costs. If this statement rings true, all self-reports of pain would therefore be conflated, resulting in a lack of differentiation between different pain aetiologies. Moreover, any untreated pain would signal poor physician practice.

## Do no harm (M.M.K. and J.D.)

We both took the Hippocratic oath and are serious about our intent to ‘do no harm’, which includes alleviating suffering. It is understandable that when pain, broadly defined as the ultimate marker of suffering, is present, the physician's responsibility/response is to provide anesthetising relief. To reduce the consequences of reliance on narcotics, we have learned that recommending non-narcotic analgesics is one important strategy, as is the discouragement of narcotic analgesics for pain management. However, the opioid conundrum may be largely over-determined for us as medical learners. The question of pain is more appropriately situated as a global exercise in understanding and managing the subjectivity of self-diagnosis inherent in the phenomenon of pain. Otherwise said, we have come to recognise that a prescription does not ensure patient adherence to medication, nor does the absence of prescriptions for narcotics mean that opioids are not widely taken for various types of somatic and psychological pain. And yet, as emerging psychiatrists, we will be charged with managing the addiction while simultaneously finding ourselves at the centre of the problem.

## Conclusions

As psychiatry learners from Uganda and Canada reflecting on the cases that brought us to the problem of the opioid crisis, we have stumbled into the reality that while we may be prescribing opioids to treat non-end-of-life pain, the use of opioids simultaneously threatens the ability of individuals to live a meaningful life. This was particularly true for Mr A. We recognise that prescribing intended to heal paradoxically contributes to the large and complex opioid problem we are likely to face throughout our careers. We have come to Jacque Derrida's notion of *pharmakon* through the experience of our clinical education.^7^ That is, the cure is also a poison. What kind of education can help us conceive of the opioid crisis where ‘pharmakon’ is a salient curriculum? The crisis is clear, but the crisis has not brought clarity for us regarding an educational response. The educational question remains: If the historical suffering inherent in everyday life is conflated with pain, whose shoulders will be broad enough to tackle the Sisyphean task?

## Data Availability

Data availability is not applicable to this article as no new data were created or analysed in this study.
